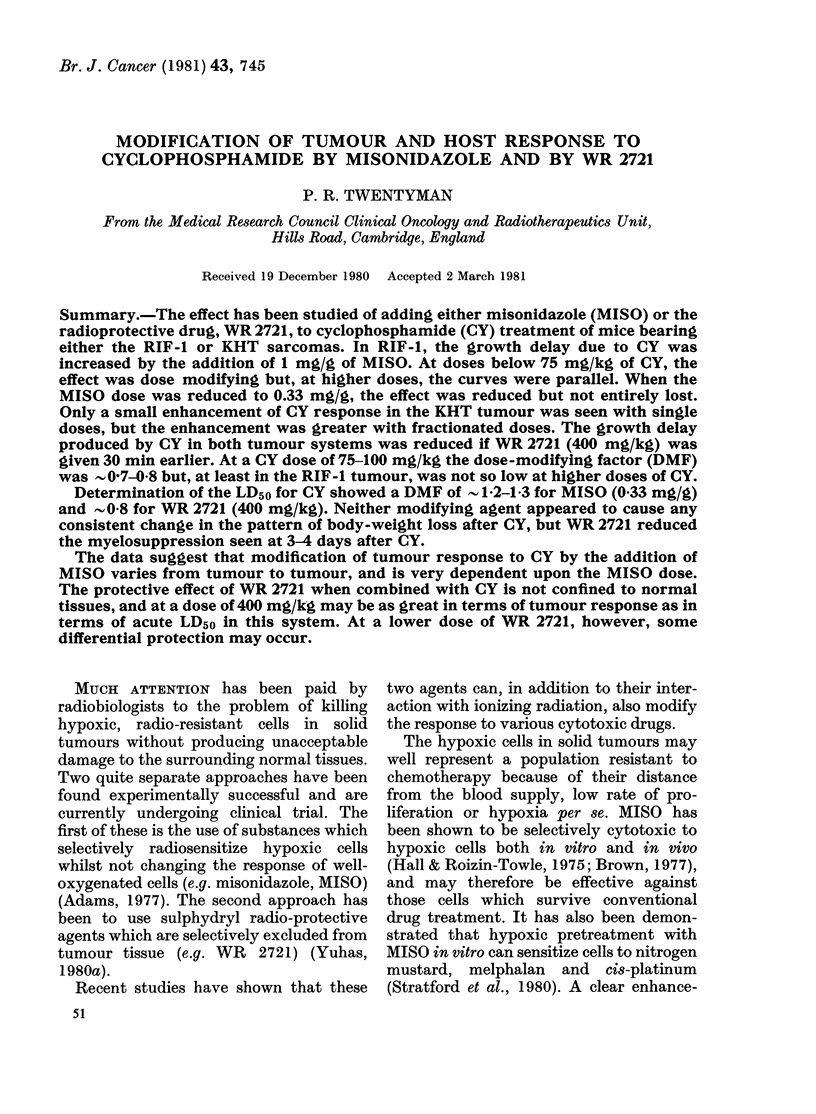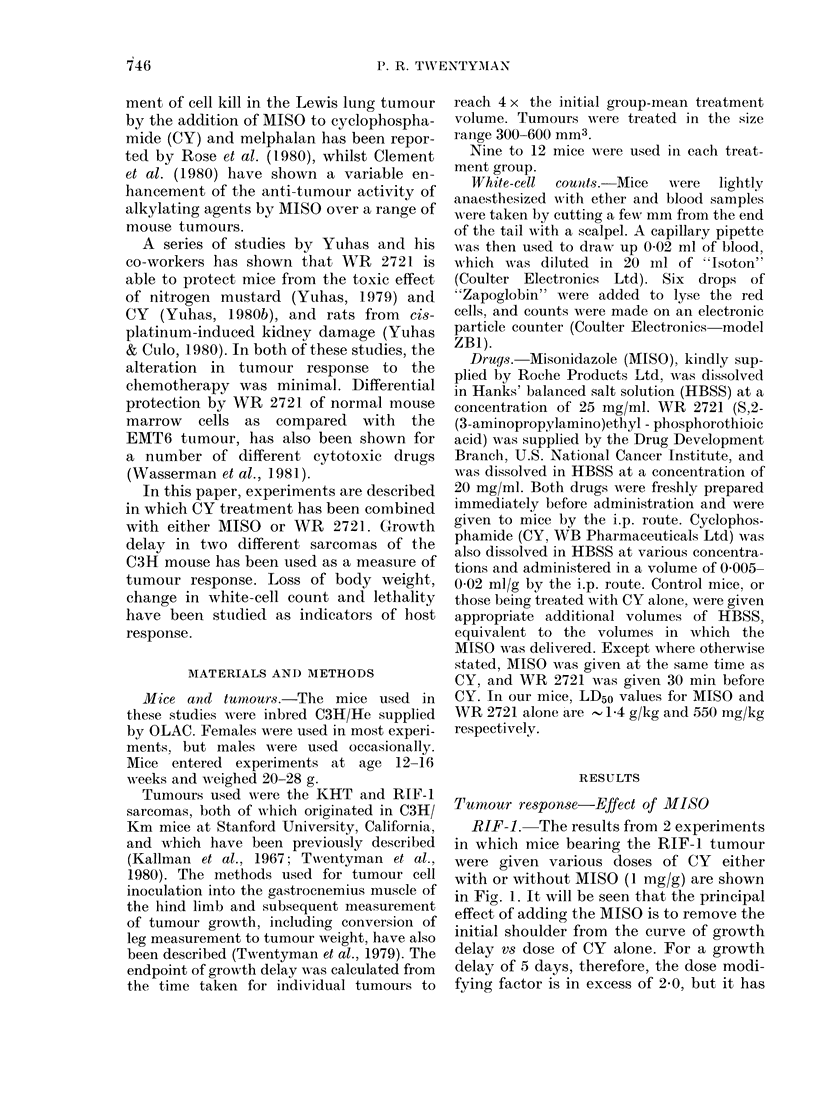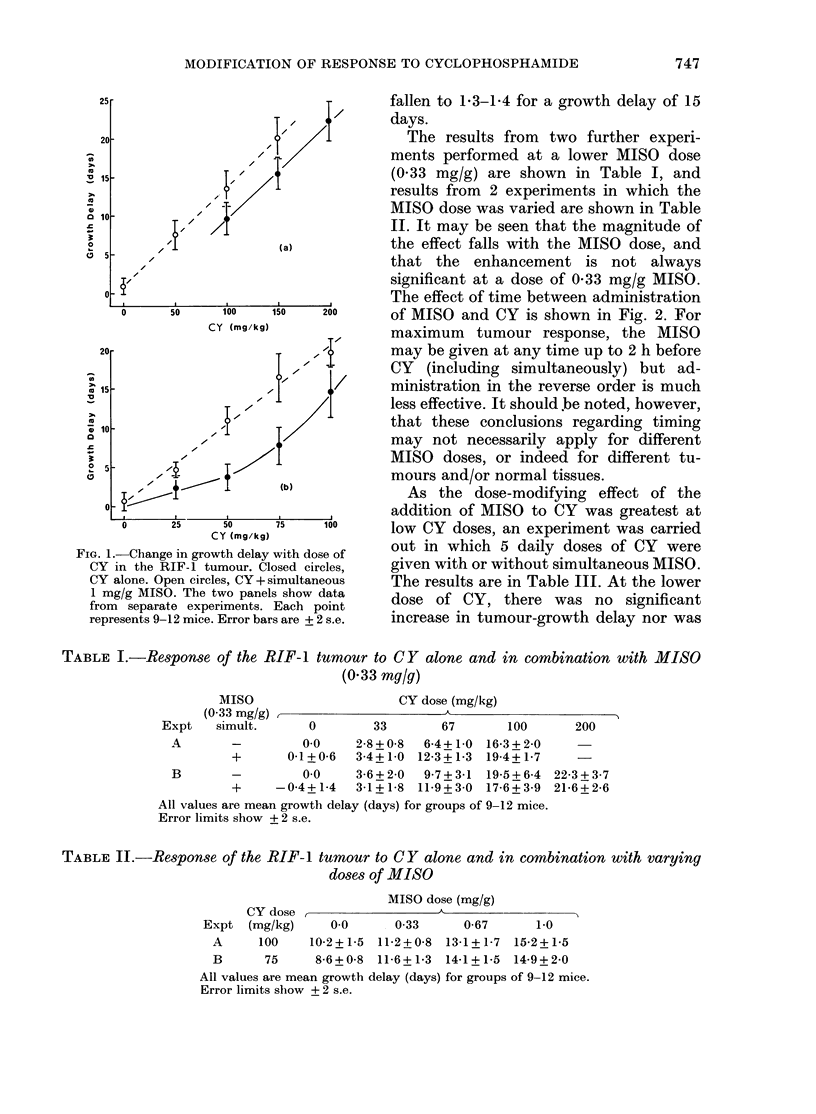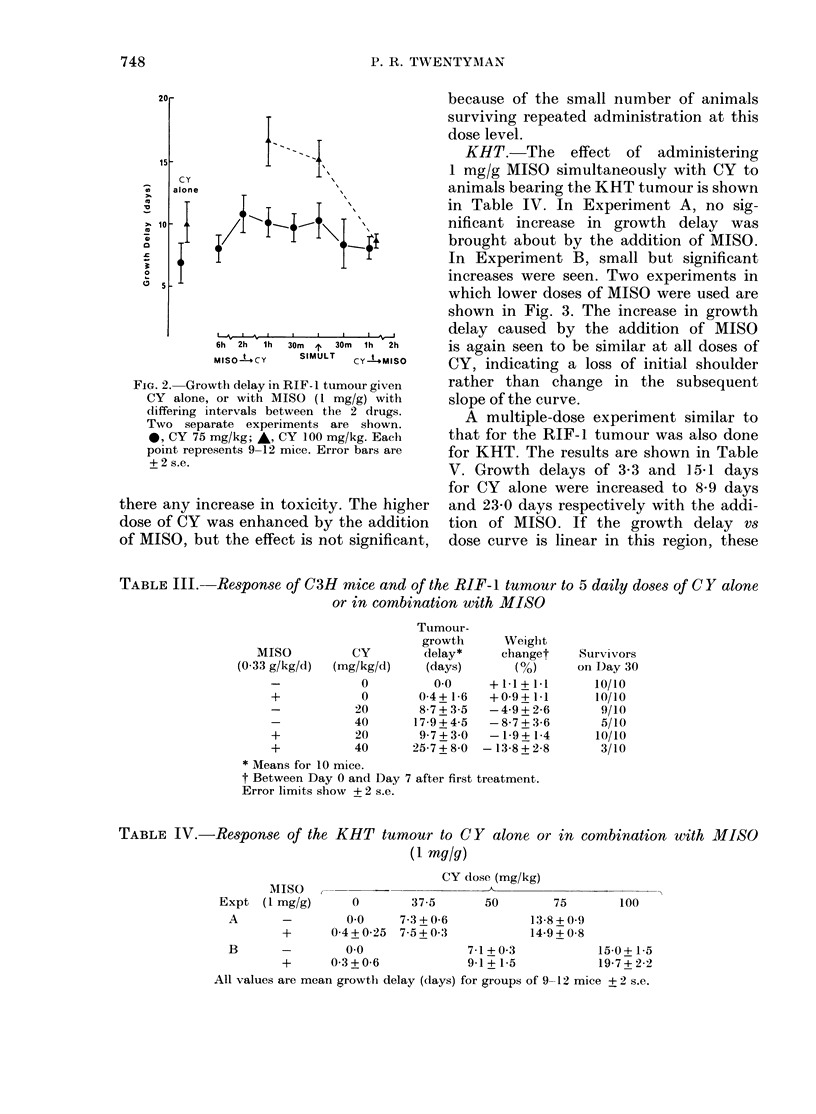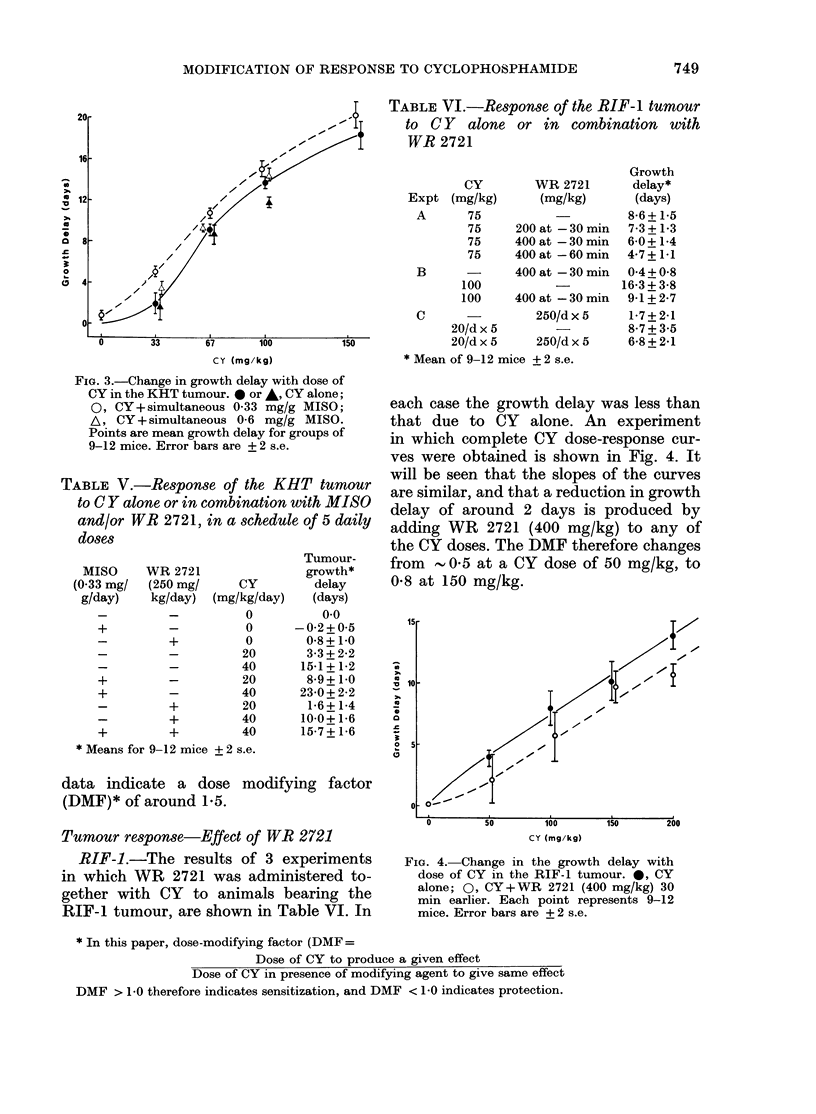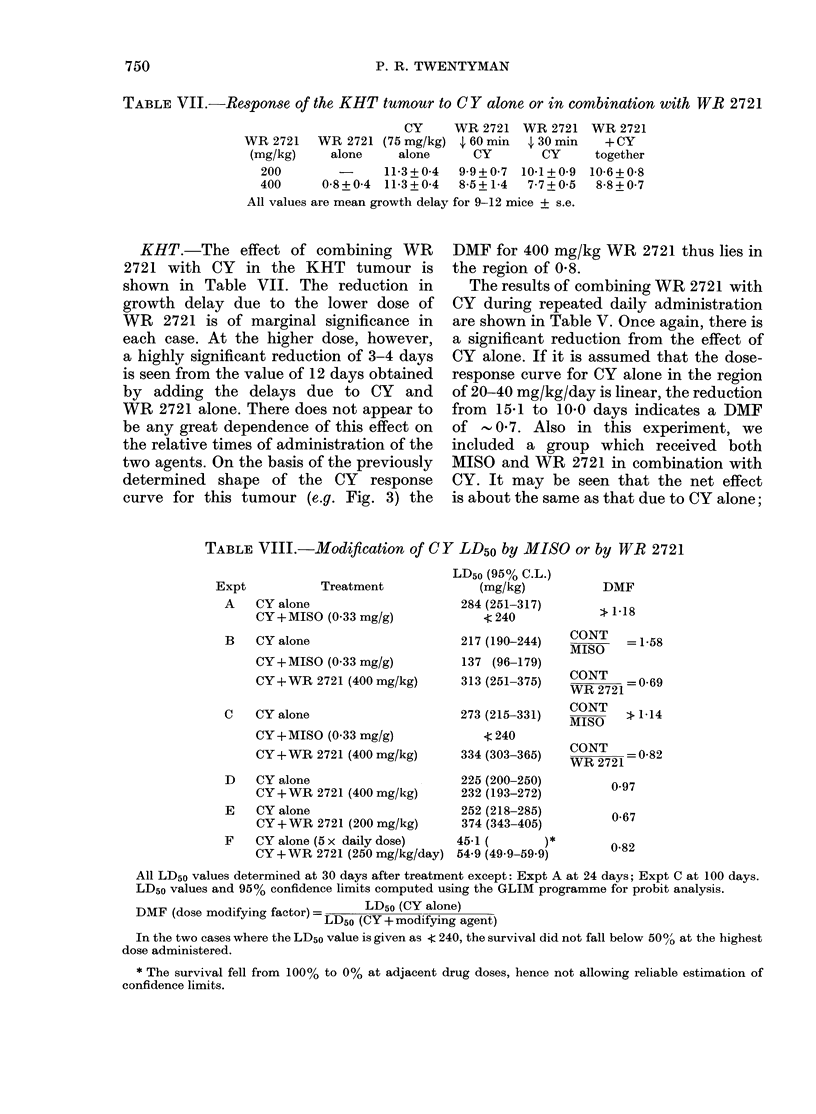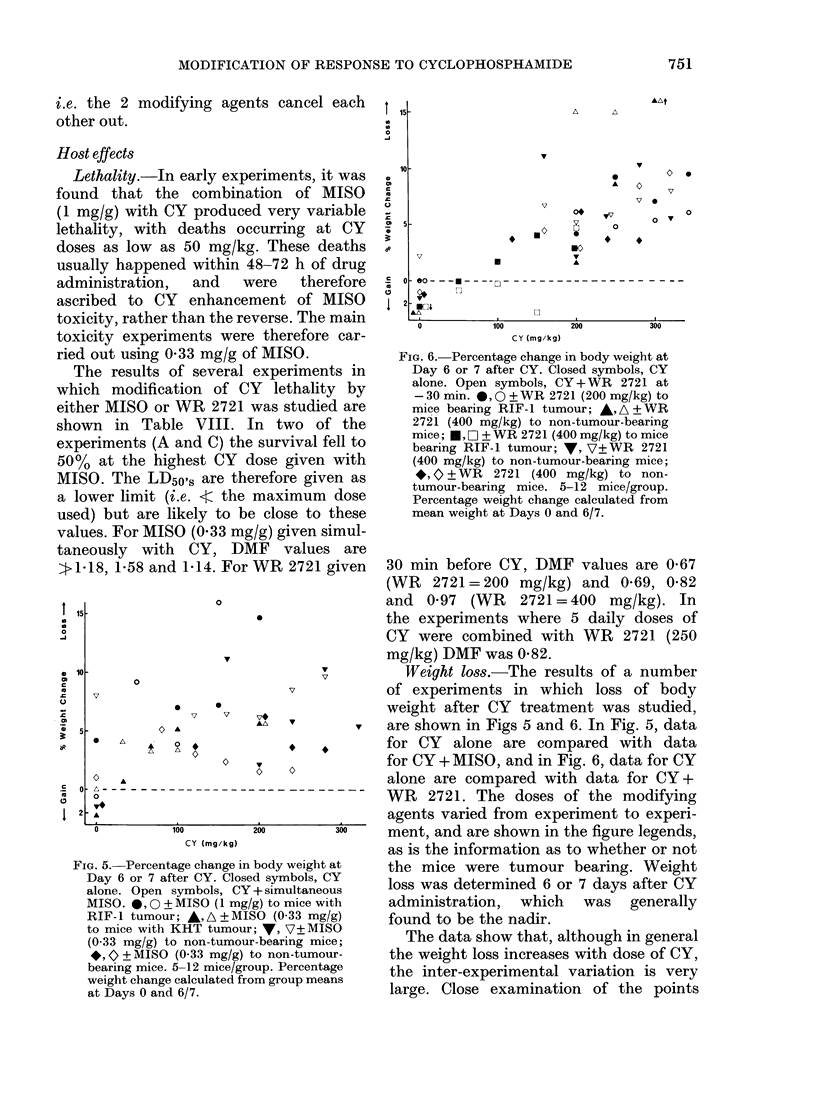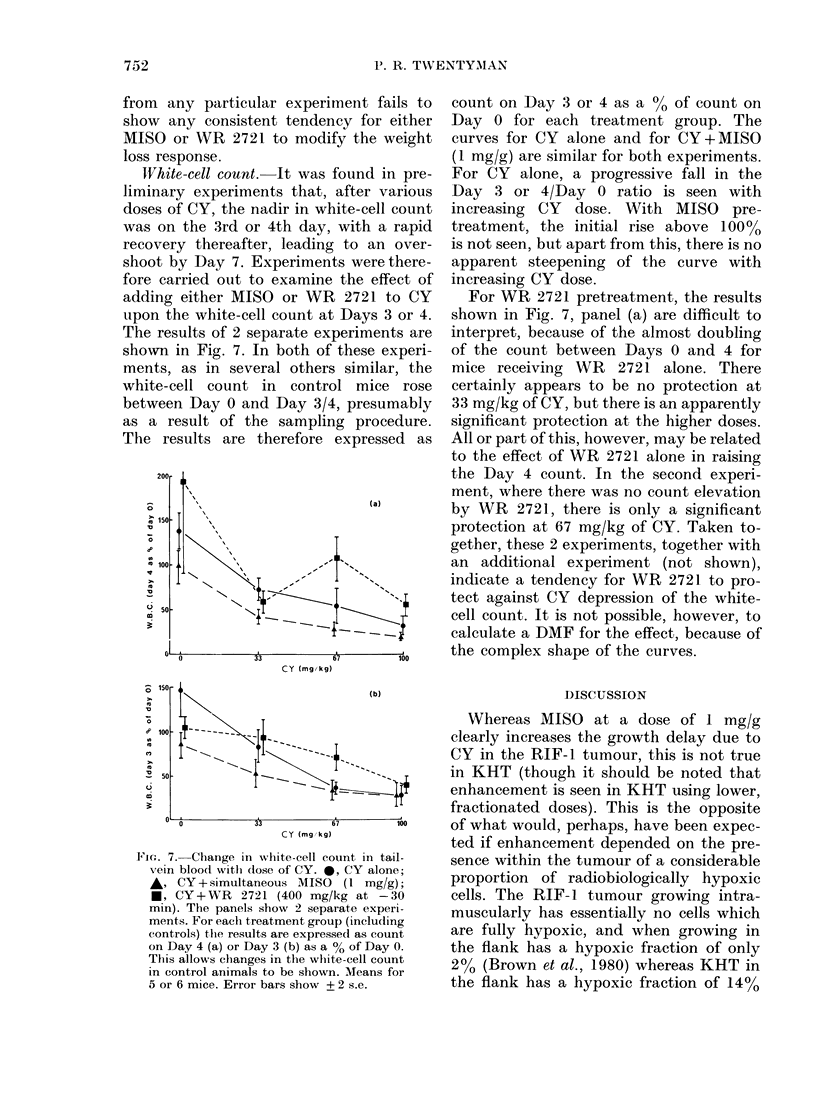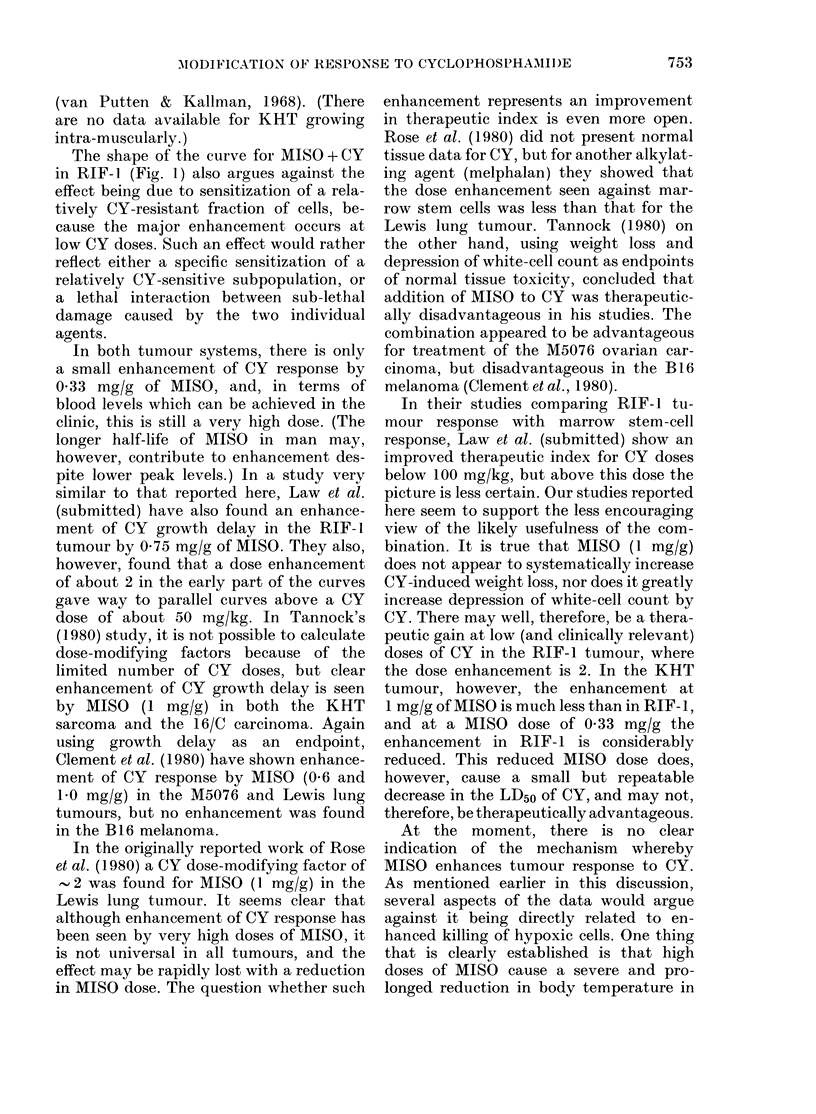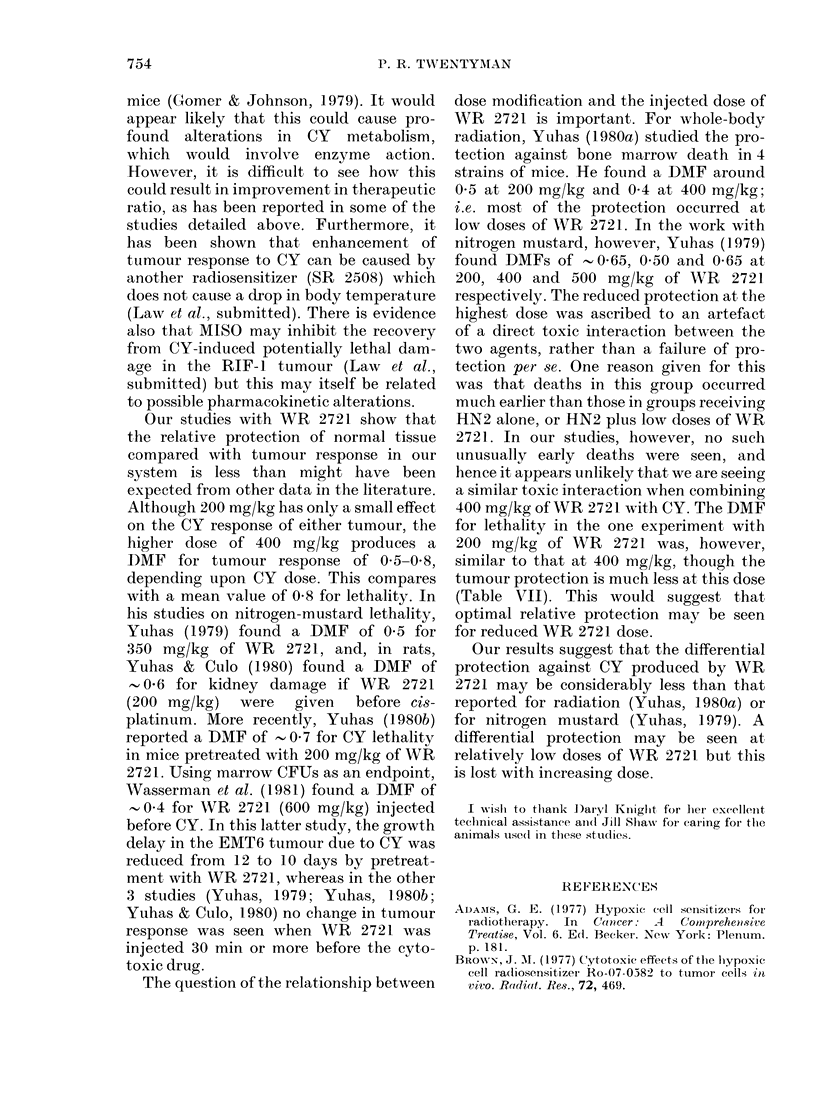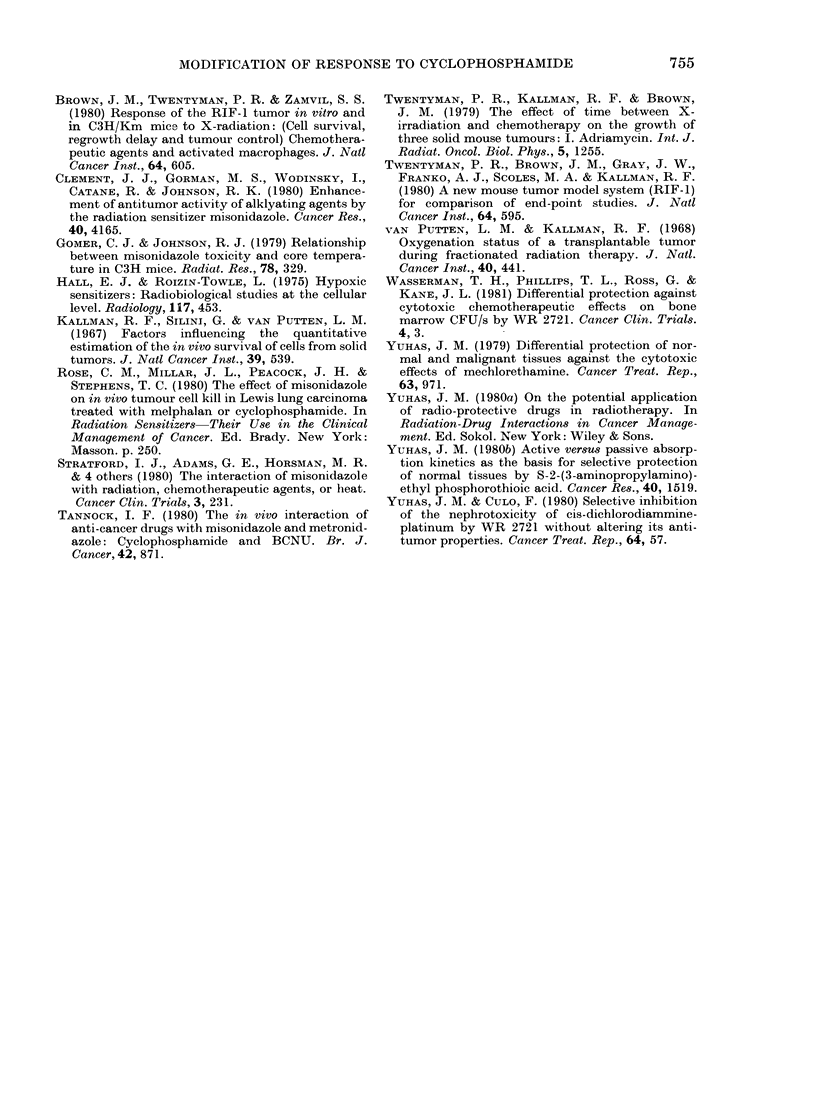# Modification of tumour and host response to cyclophosphamide by misonidazole and by WR 2721.

**DOI:** 10.1038/bjc.1981.112

**Published:** 1981-06

**Authors:** P. R. Twentyman

## Abstract

The effect has been studied of adding either misonidazole (MISO) or the radioprotective drug, WR 2721, to cyclophosphamide (CY) treatment of mice bearing either the RIF-1 or KHT sarcomas. In RIF-1, the growth delay due to CY was increased by the addition of 1 mg/g of MISO. At doses below 75 mg/kg of CY, the effect was dose modifying but, at higher doses, the curves were parallel. When the MISO dose was reduced to 0.33 mg/g, the effect was reduced but not entirely lost. Only a small enhancement of CY response in the KHT tumour was seen with single doses, but the enhancement was greater with fractionated doses. The growth delay produced by CY in both tumour systems was reduced if WR 2721 (400 mg/kg) was given 30 min earlier. At a CY dose of 75-100 mg/kg the dose-modifying factor (DMF) was approximately 0.7-0.8 but, at least in the RIF-1 tumour, was not so low at higher doses of CY. Determination of the LD50 for CY showed a DMF of approximately 1.2-1.3 for MISO (0.33 mg/g) and approximately 0.8 for WR 2721 (400 mg/kg). Neither modifying agent appeared to cause any consistent change in the pattern of body-weight loss after CY, but WR 2721 reduced the myelosuppression seen at 3-4 days after CY. The data suggest that modification of tumour response to CY by the addition of MISO varies from tumour to tumour, and is very dependent upon the MISO dose. The protective effect of WR 2721 when combined with CY is not confined to normal tissues, and at a dose of 400 mg/kg may be as great in terms of tumour response as in terms of acute LD50 in this system. At a lower dose of WR 2721, however, some differential protection may occur.


*Br. J. Cancer* (1981) **43**, 745

### MODIFICATION OF TUMOUR AND HOST RESPONSE TO CYCLOPHOSPHAMIDE BY MISONIDAZOLE AND BY WR 2721

P. R. TWENTYMAN

*From the Medical Research Council Clinical Oncology and Radiotherapeutics Unit, Hills Road, Cambridge, England*

Received 19 December 1980 Accepted 2 March 1981

**Summary.—The effect has been studied of adding either misonidazole (MISO) or the radioprotective drug, WR 2721, to cyclophosphamide (CY) treatment of mice bearing either the RIF-1 or KHT sarcomas. In RIF-1, the growth delay due to CY was increased by the addition of 1 mg/g of MISO. At doses below 75 mg/kg of CY, the effect was dose modifying but, at higher doses, the curves were parallel. When the MISO dose was reduced to 0.33 mg/g, the effect was reduced but not entirely lost. Only a small enhancement of CY response in the KHT tumour was seen with single doses, but the enhancement was greater with fractionated doses. The growth delay produced by CY in both tumour systems was reduced if WR 2721 (400 mg/kg) was given 30 min earlier. At a CY dose of 75–100 mg/kg the dose-modifying factor (DMF) was ~0·7–0·8 but, at least in the RIF-1 tumour, was not so low at higher doses of CY.**

**Determination of the $LD_{50}$ for CY showed a DMF of ~1·2–1·3 for MISO (0·33 mg/g) and ~0·8 for WR 2721 (400 mg/kg). Neither modifying agent appeared to cause any consistent change in the pattern of body-weight loss after CY, but WR 2721 reduced the myelosuppression seen at 3–4 days after CY.**

**The data suggest that modification of tumour response to CY by the addition of MISO varies from tumour to tumour, and is very dependent upon the MISO dose. The protective effect of WR 2721 when combined with CY is not confined to normal tissues, and at a dose of 400 mg/kg may be as great in terms of tumour response as in terms of acute $LD_{50}$ in this system. At a lower dose of WR 2721, however, some differential protection may occur.**

MUCH ATTENTION has been paid by radiobiologists to the problem of killing hypoxic, radio-resistant cells in solid tumours without producing unacceptable damage to the surrounding normal tissues. Two quite separate approaches have been found experimentally successful and are currently undergoing clinical trial. The first of these is the use of substances which selectively radiosensitize hypoxic cells whilst not changing the response of well-oxygenated cells (*e.g.* misonidazole, MISO) (Adams, 1977). The second approach has been to use sulphydryl radio-protective agents which are selectively excluded from tumour tissue (*e.g.* WR 2721) (Yuhas, 1980*a*).

Recent studies have shown that these two agents can, in addition to their interaction with ionizing radiation, also modify the response to various cytotoxic drugs.

The hypoxic cells in solid tumours may well represent a population resistant to chemotherapy because of their distance from the blood supply, low rate of proliferation or hypoxia *per se.* MISO has been shown to be selectively cytotoxic to hypoxic cells both *in vitro* and *in vivo* (Hall & Roizin-Towle, 1975; Brown, 1977), and may therefore be effective against those cells which survive conventional drug treatment. It has also been demonstrated that hypoxic pretreatment with MISO *in vitro* can sensitize cells to nitrogen mustard, melphalan and *cis*-platinum (Stratford *et al.*, 1980). A clear enhancement of cell kill in the Lewis lung tumour by the addition of MISO to cyclophosphamide (CY) and melphalan has been reported by Rose *et al.* (1980), whilst Clement *et al.* (1980) have shown a variable enhancement of the anti-tumour activity of alkylating agents by MISO over a range of mouse tumours.

A series of studies by Yuhas and his co-workers has shown that WR 2721 is able to protect mice from the toxic effect of nitrogen mustard (Yuhas, 1979) and CY (Yuhas, 1980*b*), and rats from *cis*-platinum-induced kidney damage (Yuhas & Culo, 1980). In both of these studies, the alteration in tumour response to the chemotherapy was minimal. Differential protection by WR 2721 of normal mouse marrow cells as compared with the EMT6 tumour, has also been shown for a number of different cytotoxic drugs (Wasserman *et al.*, 1981).

In this paper, experiments are described in which CY treatment has been combined with either MISO or WR 2721. Growth delay in two different sarcomas of the C3H mouse has been used as a measure of tumour response. Loss of body weight, change in white-cell count and lethality have been studied as indicators of host response.

### MATERIALS AND METHODS

*Mice and tumours.*—The mice used in these studies were inbred C3H/He supplied by OLAC. Females were used in most experiments, but males were used occasionally. Mice entered experiments at age 12–16 weeks and weighed 20–28 g.

Tumours used were the KHT and RIF-1 sarcomas, both of which originated in C3H/Km mice at Stanford University, California, and which have been previously described (Kallman *et al.*, 1967; Twentyman *et al.*, 1980). The methods used for tumour cell inoculation into the gastrocnemius muscle of the hind limb and subsequent measurement of tumour growth, including conversion of leg measurement to tumour weight, have also been described (Twentyman *et al.*, 1979). The endpoint of growth delay was calculated from the time taken for individual tumours to reach 4× the initial group-mean treatment volume. Tumours were treated in the size range 300–600 $mm^3$.

Nine to 12 mice were used in each treatment group.

*White-cell counts.*—Mice were lightly anaesthesized with ether and blood samples were taken by cutting a few mm from the end of the tail with a scalpel. A capillary pipette was then used to draw up 0·02 ml of blood, which was diluted in 20 ml of "Isoton" (Coulter Electronics Ltd). Six drops of "Zapoglobin" were added to lyse the red cells, and counts were made on an electronic particle counter (Coulter Electronics—model ZB1).

*Drugs.*—Misonidazole (MISO), kindly supplied by Roche Products Ltd, was dissolved in Hanks' balanced salt solution (HBSS) at a concentration of 25 mg/ml. WR 2721 (S,2-(3-aminopropylamino)ethyl - phosphorothioic acid) was supplied by the Drug Development Branch, U.S. National Cancer Institute, and was dissolved in HBSS at a concentration of 20 mg/ml. Both drugs were freshly prepared immediately before administration and were given to mice by the i.p. route. Cyclophosphamide (CY, WB Pharmaceuticals Ltd) was also dissolved in HBSS at various concentrations and administered in a volume of 0·005–0·02 ml/g by the i.p. route. Control mice, or those being treated with CY alone, were given appropriate additional volumes of HBSS, equivalent to the volumes in which the MISO was delivered. Except where otherwise stated, MISO was given at the same time as CY, and WR 2721 was given 30 min before CY. In our mice, $LD_{50}$ values for MISO and WR 2721 alone are ~1·4 g/kg and 550 mg/kg respectively.

### RESULTS

### *Tumour response—Effect of MISO*

*RIF-1.*—The results from 2 experiments in which mice bearing the RIF-1 tumour were given various doses of CY either with or without MISO (1 mg/g) are shown in Fig. 1. It will be seen that the principal effect of adding the MISO is to remove the initial shoulder from the curve of growth delay *vs* dose of CY alone. For a growth delay of 5 days, therefore, the dose modifying factor is in excess of 2·0, but it has

FIG. 1.—Change in growth delay with dose of CY in the RIF-1 tumour. Closed circles, CY alone. Open circles, CY + simultaneous 1 mg/g MISO. The two panels show data from separate experiments. Each point represents 9–12 mice. Error bars are ±2 s.e.

fallen to 1·3–1·4 for a growth delay of 15 days.

The results from two further experiments performed at a lower MISO dose (0·33 mg/g) are shown in Table I, and results from 2 experiments in which the MISO dose was varied are shown in Table II. It may be seen that the magnitude of the effect falls with the MISO dose, and that the enhancement is not always significant at a dose of 0·33 mg/g MISO. The effect of time between administration of MISO and CY is shown in Fig. 2. For maximum tumour response, the MISO may be given at any time up to 2 h before CY (including simultaneously) but administration in the reverse order is much less effective. It should be noted, however, that these conclusions regarding timing may not necessarily apply for different MISO doses, or indeed for different tumours and/or normal tissues.

As the dose-modifying effect of the addition of MISO to CY was greatest at low CY doses, an experiment was carried out in which 5 daily doses of CY were given with or without simultaneous MISO. The results are in Table III. At the lower dose of CY, there was no significant increase in tumour-growth delay nor was

TABLE I.—*Response of the RIF-1 tumour to CY alone and in combination with MISO (0·33 mg/g)*

| Expt | MISO (0·33 mg/g) simult. | CY dose (mg/kg) 0 | 33 | 67 | 100 | 200 |
|---|---|---|---|---|---|---|
| A | − | 0·0 | 2·8±0·8 | 6·4±1·0 | 16·3±2·0 | — |
| | + | 0·1±0·6 | 3·4±1·0 | 12·3±1·3 | 19·4±1·7 | — |
| B | − | 0·0 | 3·6±2·0 | 9·7±3·1 | 19·5±6·4 | 22·3±3·7 |
| | + | −0·4±1·4 | 3·1±1·8 | 11·9±3·0 | 17·6±3·9 | 21·6±2·6 |

All values are mean growth delay (days) for groups of 9–12 mice.
Error limits show ±2 s.e.

TABLE II.—*Response of the RIF-1 tumour to CY alone and in combination with varying doses of MISO*

| Expt | CY dose (mg/kg) | MISO dose (mg/g) 0·0 | 0·33 | 0·67 | 1·0 |
|---|---|---|---|---|---|
| A | 100 | 10·2±1·5 | 11·2±0·8 | 13·1±1·7 | 15·2±1·5 |
| B | 75 | 8·6±0·8 | 11·6±1·3 | 14·1±1·5 | 14·9±2·0 |

All values are mean growth delay (days) for groups of 9–12 mice.
Error limits show ±2 s.e.

FIG. 2.—Growth delay in RIF-1 tumour given CY alone, or with MISO (1 mg/g) with differing intervals between the 2 drugs. Two separate experiments are shown. ●, CY 75 mg/kg; ▲, CY 100 mg/kg. Each point represents 9–12 mice. Error bars are ±2 s.e.

there any increase in toxicity. The higher dose of CY was enhanced by the addition of MISO, but the effect is not significant, because of the small number of animals surviving repeated administration at this dose level.

*KHT*.—The effect of administering 1 mg/g MISO simultaneously with CY to animals bearing the KHT tumour is shown in Table IV. In Experiment A, no significant increase in growth delay was brought about by the addition of MISO. In Experiment B, small but significant increases were seen. Two experiments in which lower doses of MISO were used are shown in Fig. 3. The increase in growth delay caused by the addition of MISO is again seen to be similar at all doses of CY, indicating a loss of initial shoulder rather than change in the subsequent slope of the curve.

A multiple-dose experiment similar to that for the RIF-1 tumour was also done for KHT. The results are shown in Table V. Growth delays of 3·3 and 15·1 days for CY alone were increased to 8·9 days and 23·0 days respectively with the addition of MISO. If the growth delay *vs* dose curve is linear in this region, these

TABLE III.—*Response of C3H mice and of the RIF-1 tumour to 5 daily doses of CY alone or in combination with MISO*

| MISO (0·33 g/kg/d) | CY (mg/kg/d) | Tumour-growth delay* (days) | Weight change† (%) | Survivors on Day 30 |
|---|---|---|---|---|
| – | 0 | 0·0 | +1·1±1·1 | 10/10 |
| + | 0 | 0·4±1·6 | +0·9±1·1 | 10/10 |
| – | 20 | 8·7±3·5 | −4·9±2·6 | 9/10 |
| – | 40 | 17·9±4·5 | −8·7±3·6 | 5/10 |
| + | 20 | 9·7±3·0 | −1·9±1·4 | 10/10 |
| + | 40 | 25·7±8·0 | −13·8±2·8 | 3/10 |

* Means for 10 mice.
† Between Day 0 and Day 7 after first treatment.
Error limits show ±2 s.e.

TABLE IV.—*Response of the KHT tumour to CY alone or in combination with MISO (1 mg/g)*

| Expt | MISO (1 mg/g) | CY dose (mg/kg) 0 | 37·5 | 50 | 75 | 100 |
|---|---|---|---|---|---|---|
| A | – | 0·0 | 7·3±0·6 | | 13·8±0·9 | |
| | + | 0·4±0·25 | 7·5±0·3 | | 14·9±0·8 | |
| B | – | 0·0 | | 7·1±0·3 | | 15·0±1·5 |
| | + | 0·3±0·6 | | 9·1±1·5 | | 19·7±2·2 |

All values are mean growth delay (days) for groups of 9–12 mice ±2 s.e.

FIG. 3.—Change in growth delay with dose of CY in the KHT tumour. ● or ▲, CY alone; ○, CY+simultaneous 0·33 mg/g MISO; △, CY+simultaneous 0·6 mg/g MISO. Points are mean growth delay for groups of 9–12 mice. Error bars are ±2 s.e.

TABLE V.—*Response of the KHT tumour to CY alone or in combination with MISO and/or WR 2721, in a schedule of 5 daily doses*

| MISO (0·33 mg/g/day) | WR 2721 (250 mg/kg/day) | CY (mg/kg/day) | Tumour-growth* delay (days) |
|---|---|---|---|
| – | – | 0 | 0·0 |
| + | – | 0 | −0·2±0·5 |
| – | + | 0 | 0·8±1·0 |
| – | – | 20 | 3·3±2·2 |
| – | – | 40 | 15·1±1·2 |
| + | – | 20 | 8·9±1·0 |
| + | – | 40 | 23·0±2·2 |
| – | + | 20 | 1·6±1·4 |
| – | + | 40 | 10·0±1·6 |
| + | + | 40 | 15·7±1·6 |

* Means for 9–12 mice ±2 s.e.

data indicate a dose modifying factor (DMF)* of around 1·5.

*Tumour response—Effect of WR 2721*

*RIF-1*.—The results of 3 experiments in which WR 2721 was administered together with CY to animals bearing the RIF-1 tumour, are shown in Table VI. In each case the growth delay was less than that due to CY alone. An experiment in which complete CY dose-response curves were obtained is shown in Fig. 4. It will be seen that the slopes of the curves are similar, and that a reduction in growth delay of around 2 days is produced by adding WR 2721 (400 mg/kg) to any of the CY doses. The DMF therefore changes from ~0·5 at a CY dose of 50 mg/kg, to 0·8 at 150 mg/kg.

TABLE VI.—*Response of the RIF-1 tumour to CY alone or in combination with WR 2721*

| Expt | CY (mg/kg) | WR 2721 (mg/kg) | Growth delay* (days) |
|---|---|---|---|
| A | 75 | — | 8·6±1·5 |
| | 75 | 200 at −30 min | 7·3±1·3 |
| | 75 | 400 at −30 min | 6·0±1·4 |
| | 75 | 400 at −60 min | 4·7±1·1 |
| B | — | 400 at −30 min | 0·4±0·8 |
| | 100 | — | 16·3±3·8 |
| | 100 | 400 at −30 min | 9·1±2·7 |
| C | — | 250/d × 5 | 1·7±2·1 |
| | 20/d × 5 | — | 8·7±3·5 |
| | 20/d × 5 | 250/d × 5 | 6·8±2·1 |

* Mean of 9–12 mice ±2 s.e.

FIG. 4.—Change in the growth delay with dose of CY in the RIF-1 tumour. ●, CY alone; ○, CY+WR 2721 (400 mg/kg) 30 min earlier. Each point represents 9–12 mice. Error bars are ±2 s.e.

* In this paper, dose-modifying factor (DMF = $\frac{\text{Dose of CY to produce a given effect}}{\text{Dose of CY in presence of modifying agent to give same effect}}$

DMF > 1·0 therefore indicates sensitization, and DMF < 1·0 indicates protection.

TABLE VII.—*Response of the KHT tumour to CY alone or in combination with WR* 2721

| WR 2721 (mg/kg) | WR 2721 alone | CY (75 mg/kg) alone | WR 2721 ↓ 60 min CY | WR 2721 ↓ 30 min CY | WR 2721 + CY together |
|---|---|---|---|---|---|
| 200 | — | 11·3 ± 0·4 | 9·9 ± 0·7 | 10·1 ± 0·9 | 10·6 ± 0·8 |
| 400 | 0·8 ± 0·4 | 11·3 ± 0·4 | 8·5 ± 1·4 | 7·7 ± 0·5 | 8·8 ± 0·7 |

All values are mean growth delay for 9–12 mice ± s.e.

*KHT*.—The effect of combining WR 2721 with CY in the KHT tumour is shown in Table VII. The reduction in growth delay due to the lower dose of WR 2721 is of marginal significance in each case. At the higher dose, however, a highly significant reduction of 3–4 days is seen from the value of 12 days obtained by adding the delays due to CY and WR 2721 alone. There does not appear to be any great dependence of this effect on the relative times of administration of the two agents. On the basis of the previously determined shape of the CY response curve for this tumour (*e.g.* Fig. 3) the DMF for 400 mg/kg WR 2721 thus lies in the region of 0·8.

The results of combining WR 2721 with CY during repeated daily administration are shown in Table V. Once again, there is a significant reduction from the effect of CY alone. If it is assumed that the dose-response curve for CY alone in the region of 20–40 mg/kg/day is linear, the reduction from 15·1 to 10·0 days indicates a DMF of ~0·7. Also in this experiment, we included a group which received both MISO and WR 2721 in combination with CY. It may be seen that the net effect is about the same as that due to CY alone;

TABLE VIII.—*Modification of CY* $LD_{50}$ *by MISO or by WR* 2721

| Expt | Treatment | $LD_{50}$ (95% C.L.) (mg/kg) | DMF |
|---|---|---|---|
| A | CY alone | 284 (251–317) | ≯ 1·18 |
| | CY + MISO (0·33 mg/g) | ≮ 240 | |
| B | CY alone | 217 (190–244) | CONT/MISO = 1·58 |
| | CY + MISO (0·33 mg/g) | 137 (96–179) | |
| | CY + WR 2721 (400 mg/kg) | 313 (251–375) | CONT/WR 2721 = 0·69 |
| C | CY alone | 273 (215–331) | CONT/MISO ≯ 1·14 |
| | CY + MISO (0·33 mg/g) | ≮ 240 | |
| | CY + WR 2721 (400 mg/kg) | 334 (303–365) | CONT/WR 2721 = 0·82 |
| D | CY alone | 225 (200–250) | 0·97 |
| | CY + WR 2721 (400 mg/kg) | 232 (193–272) | |
| E | CY alone | 252 (218–285) | 0·67 |
| | CY + WR 2721 (200 mg/kg) | 374 (343–405) | |
| F | CY alone (5 × daily dose) | 45·1 ( )* | 0·82 |
| | CY + WR 2721 (250 mg/kg/day) | 54·9 (49·9–59·9) | |

All $LD_{50}$ values determined at 30 days after treatment except: Expt A at 24 days; Expt C at 100 days. $LD_{50}$ values and 95% confidence limits computed using the GLIM programme for probit analysis.

$$\text{DMF (dose modifying factor)} = \frac{LD_{50}\text{ (CY alone)}}{LD_{50}\text{ (CY + modifying agent)}}$$

In the two cases where the $LD_{50}$ value is given as ≮ 240, the survival did not fall below 50% at the highest dose administered.

* The survival fell from 100% to 0% at adjacent drug doses, hence not allowing reliable estimation of confidence limits.

*i.e.* the 2 modifying agents cancel each other out.

### *Host effects*

*Lethality.*—In early experiments, it was found that the combination of MISO (1 mg/g) with CY produced very variable lethality, with deaths occurring at CY doses as low as 50 mg/kg. These deaths usually happened within 48–72 h of drug administration, and were therefore ascribed to CY enhancement of MISO toxicity, rather than the reverse. The main toxicity experiments were therefore carried out using 0·33 mg/g of MISO.

The results of several experiments in which modification of CY lethality by either MISO or WR 2721 was studied are shown in Table VIII. In two of the experiments (A and C) the survival fell to 50% at the highest CY dose given with MISO. The $LD_{50}$'s are therefore given as a lower limit (*i.e.* $\nless$ the maximum dose used) but are likely to be close to these values. For MISO (0·33 mg/g) given simultaneously with CY, DMF values are $\ngtr$1·18, 1·58 and 1·14. For WR 2721 given

FIG. 5.—Percentage change in body weight at Day 6 or 7 after CY. Closed symbols, CY alone. Open symbols, CY + simultaneous MISO. ●, ○ ±MISO (1 mg/g) to mice with RIF-1 tumour; ▲, △ ±MISO (0·33 mg/g) to mice with KHT tumour; ▼, ▽±MISO (0·33 mg/g) to non-tumour-bearing mice; ◆, ◇ ±MISO (0·33 mg/g) to non-tumour-bearing mice. 5–12 mice/group. Percentage weight change calculated from group means at Days 0 and 6/7.

FIG. 6.—Percentage change in body weight at Day 6 or 7 after CY. Closed symbols, CY alone. Open symbols, CY + WR 2721 at −30 min. ●, ○ ±WR 2721 (200 mg/kg) to mice bearing RIF-1 tumour; ▲, △ ±WR 2721 (400 mg/kg) to non-tumour-bearing mice; ■, □ ±WR 2721 (400 mg/kg) to mice bearing RIF-1 tumour; ▼, ▽±WR 2721 (400 mg/kg) to non-tumour-bearing mice; ◆, ◇ ±WR 2721 (400 mg/kg) to non-tumour-bearing mice. 5–12 mice/group. Percentage weight change calculated from mean weight at Days 0 and 6/7.

30 min before CY, DMF values are 0·67 (WR 2721 = 200 mg/kg) and 0·69, 0·82 and 0·97 (WR 2721 = 400 mg/kg). In the experiments where 5 daily doses of CY were combined with WR 2721 (250 mg/kg) DMF was 0·82.

*Weight loss.*—The results of a number of experiments in which loss of body weight after CY treatment was studied, are shown in Figs 5 and 6. In Fig. 5, data for CY alone are compared with data for CY + MISO, and in Fig. 6, data for CY alone are compared with data for CY + WR 2721. The doses of the modifying agents varied from experiment to experiment, and are shown in the figure legends, as is the information as to whether or not the mice were tumour bearing. Weight loss was determined 6 or 7 days after CY administration, which was generally found to be the nadir.

The data show that, although in general the weight loss increases with dose of CY, the inter-experimental variation is very large. Close examination of the points from any particular experiment fails to show any consistent tendency for either MISO or WR 2721 to modify the weight loss response.

*White-cell count.*—It was found in preliminary experiments that, after various doses of CY, the nadir in white-cell count was on the 3rd or 4th day, with a rapid recovery thereafter, leading to an overshoot by Day 7. Experiments were therefore carried out to examine the effect of adding either MISO or WR 2721 to CY upon the white-cell count at Days 3 or 4. The results of 2 separate experiments are shown in Fig. 7. In both of these experiments, as in several others similar, the white-cell count in control mice rose between Day 0 and Day 3/4, presumably as a result of the sampling procedure. The results are therefore expressed as

FIG. 7.—Change in white-cell count in tail-vein blood with dose of CY. ●, CY alone; ▲, CY+simultaneous MISO (1 mg/g); ■, CY+WR 2721 (400 mg/kg at −30 min). The panels show 2 separate experiments. For each treatment group (including controls) the results are expressed as count on Day 4 (a) or Day 3 (b) as a % of Day 0. This allows changes in the white-cell count in control animals to be shown. Means for 5 or 6 mice. Error bars show ±2 s.e.

count on Day 3 or 4 as a % of count on Day 0 for each treatment group. The curves for CY alone and for CY+MISO (1 mg/g) are similar for both experiments. For CY alone, a progressive fall in the Day 3 or 4/Day 0 ratio is seen with increasing CY dose. With MISO pretreatment, the initial rise above 100% is not seen, but apart from this, there is no apparent steepening of the curve with increasing CY dose.

For WR 2721 pretreatment, the results shown in Fig. 7, panel (a) are difficult to interpret, because of the almost doubling of the count between Days 0 and 4 for mice receiving WR 2721 alone. There certainly appears to be no protection at 33 mg/kg of CY, but there is an apparently significant protection at the higher doses. All or part of this, however, may be related to the effect of WR 2721 alone in raising the Day 4 count. In the second experiment, where there was no count elevation by WR 2721, there is only a significant protection at 67 mg/kg of CY. Taken together, these 2 experiments, together with an additional experiment (not shown), indicate a tendency for WR 2721 to protect against CY depression of the white-cell count. It is not possible, however, to calculate a DMF for the effect, because of the complex shape of the curves.

### DISCUSSION

Whereas MISO at a dose of 1 mg/g clearly increases the growth delay due to CY in the RIF-1 tumour, this is not true in KHT (though it should be noted that enhancement is seen in KHT using lower, fractionated doses). This is the opposite of what would, perhaps, have been expected if enhancement depended on the presence within the tumour of a considerable proportion of radiobiologically hypoxic cells. The RIF-1 tumour growing intramuscularly has essentially no cells which are fully hypoxic, and when growing in the flank has a hypoxic fraction of only 2% (Brown *et al.*, 1980) whereas KHT in the flank has a hypoxic fraction of 14%

(van Putten & Kallman, 1968). (There are no data available for KHT growing intra-muscularly.)

The shape of the curve for MISO + CY in RIF-1 (Fig. 1) also argues against the effect being due to sensitization of a relatively CY-resistant fraction of cells, because the major enhancement occurs at low CY doses. Such an effect would rather reflect either a specific sensitization of a relatively CY-sensitive subpopulation, or a lethal interaction between sub-lethal damage caused by the two individual agents.

In both tumour systems, there is only a small enhancement of CY response by 0·33 mg/g of MISO, and, in terms of blood levels which can be achieved in the clinic, this is still a very high dose. (The longer half-life of MISO in man may, however, contribute to enhancement despite lower peak levels.) In a study very similar to that reported here, Law *et al.* (submitted) have also found an enhancement of CY growth delay in the RIF-1 tumour by 0·75 mg/g of MISO. They also, however, found that a dose enhancement of about 2 in the early part of the curves gave way to parallel curves above a CY dose of about 50 mg/kg. In Tannock's (1980) study, it is not possible to calculate dose-modifying factors because of the limited number of CY doses, but clear enhancement of CY growth delay is seen by MISO (1 mg/g) in both the KHT sarcoma and the 16/C carcinoma. Again using growth delay as an endpoint, Clement *et al.* (1980) have shown enhancement of CY response by MISO (0·6 and 1·0 mg/g) in the M5076 and Lewis lung tumours, but no enhancement was found in the B16 melanoma.

In the originally reported work of Rose *et al.* (1980) a CY dose-modifying factor of ~2 was found for MISO (1 mg/g) in the Lewis lung tumour. It seems clear that although enhancement of CY response has been seen by very high doses of MISO, it is not universal in all tumours, and the effect may be rapidly lost with a reduction in MISO dose. The question whether such enhancement represents an improvement in therapeutic index is even more open. Rose *et al.* (1980) did not present normal tissue data for CY, but for another alkylating agent (melphalan) they showed that the dose enhancement seen against marrow stem cells was less than that for the Lewis lung tumour. Tannock (1980) on the other hand, using weight loss and depression of white-cell count as endpoints of normal tissue toxicity, concluded that addition of MISO to CY was therapeutically disadvantageous in his studies. The combination appeared to be advantageous for treatment of the M5076 ovarian carcinoma, but disadvantageous in the B16 melanoma (Clement *et al.*, 1980).

In their studies comparing RIF-1 tumour response with marrow stem-cell response, Law *et al.* (submitted) show an improved therapeutic index for CY doses below 100 mg/kg, but above this dose the picture is less certain. Our studies reported here seem to support the less encouraging view of the likely usefulness of the combination. It is true that MISO (1 mg/g) does not appear to systematically increase CY-induced weight loss, nor does it greatly increase depression of white-cell count by CY. There may well, therefore, be a therapeutic gain at low (and clinically relevant) doses of CY in the RIF-1 tumour, where the dose enhancement is 2. In the KHT tumour, however, the enhancement at 1 mg/g of MISO is much less than in RIF-1, and at a MISO dose of 0·33 mg/g the enhancement in RIF-1 is considerably reduced. This reduced MISO dose does, however, cause a small but repeatable decrease in the $LD_{50}$ of CY, and may not, therefore, be therapeutically advantageous.

At the moment, there is no clear indication of the mechanism whereby MISO enhances tumour response to CY. As mentioned earlier in this discussion, several aspects of the data would argue against it being directly related to enhanced killing of hypoxic cells. One thing that is clearly established is that high doses of MISO cause a severe and prolonged reduction in body temperature in mice (Gomer & Johnson, 1979). It would appear likely that this could cause profound alterations in CY metabolism, which would involve enzyme action. However, it is difficult to see how this could result in improvement in therapeutic ratio, as has been reported in some of the studies detailed above. Furthermore, it has been shown that enhancement of tumour response to CY can be caused by another radiosensitizer (SR 2508) which does not cause a drop in body temperature (Law *et al.*, submitted). There is evidence also that MISO may inhibit the recovery from CY-induced potentially lethal damage in the RIF-1 tumour (Law *et al.*, submitted) but this may itself be related to possible pharmacokinetic alterations.

Our studies with WR 2721 show that the relative protection of normal tissue compared with tumour response in our system is less than might have been expected from other data in the literature. Although 200 mg/kg has only a small effect on the CY response of either tumour, the higher dose of 400 mg/kg produces a DMF for tumour response of 0·5–0·8, depending upon CY dose. This compares with a mean value of 0·8 for lethality. In his studies on nitrogen-mustard lethality, Yuhas (1979) found a DMF of 0·5 for 350 mg/kg of WR 2721, and, in rats, Yuhas & Culo (1980) found a DMF of ~0·6 for kidney damage if WR 2721 (200 mg/kg) were given before *cis*-platinum. More recently, Yuhas (1980*b*) reported a DMF of ~0·7 for CY lethality in mice pretreated with 200 mg/kg of WR 2721. Using marrow CFUs as an endpoint, Wasserman *et al.* (1981) found a DMF of ~0·4 for WR 2721 (600 mg/kg) injected before CY. In this latter study, the growth delay in the EMT6 tumour due to CY was reduced from 12 to 10 days by pretreatment with WR 2721, whereas in the other 3 studies (Yuhas, 1979; Yuhas, 1980*b*; Yuhas & Culo, 1980) no change in tumour response was seen when WR 2721 was injected 30 min or more before the cytotoxic drug.

The question of the relationship between dose modification and the injected dose of WR 2721 is important. For whole-body radiation, Yuhas (1980*a*) studied the protection against bone marrow death in 4 strains of mice. He found a DMF around 0·5 at 200 mg/kg and 0·4 at 400 mg/kg; *i.e.* most of the protection occurred at low doses of WR 2721. In the work with nitrogen mustard, however, Yuhas (1979) found DMFs of ~0·65, 0·50 and 0·65 at 200, 400 and 500 mg/kg of WR 2721 respectively. The reduced protection at the highest dose was ascribed to an artefact of a direct toxic interaction between the two agents, rather than a failure of protection *per se*. One reason given for this was that deaths in this group occurred much earlier than those in groups receiving HN2 alone, or HN2 plus low doses of WR 2721. In our studies, however, no such unusually early deaths were seen, and hence it appears unlikely that we are seeing a similar toxic interaction when combining 400 mg/kg of WR 2721 with CY. The DMF for lethality in the one experiment with 200 mg/kg of WR 2721 was, however, similar to that at 400 mg/kg, though the tumour protection is much less at this dose (Table VII). This would suggest that optimal relative protection may be seen for reduced WR 2721 dose.

Our results suggest that the differential protection against CY produced by WR 2721 may be considerably less than that reported for radiation (Yuhas, 1980*a*) or for nitrogen mustard (Yuhas, 1979). A differential protection may be seen at relatively low doses of WR 2721 but this is lost with increasing dose.

I wish to thank Daryl Knight for her excellent technical assistance and Jill Shaw for caring for the animals used in these studies.

### REFERENCES

ADAMS, G. E. (1977) Hypoxic cell sensitizers for radiotherapy. In *Cancer: A Comprehensive Treatise*, Vol. 6. Ed. Becker. New York: Plenum. p. 181.

BROWN, J. M. (1977) Cytotoxic effects of the hypoxic cell radiosensitizer Ro-07-0582 to tumor cells *in vivo*. *Radiat. Res.*, **72**, 469.

Brown, J. M., Twentyman, P. R. & Zamvil, S. S. (1980) Response of the RIF-1 tumor *in vitro* and in C3H/Km mice to X-radiation: (Cell survival, regrowth delay and tumour control) Chemotherapeutic agents and activated macrophages. *J. Natl Cancer Inst.*, **64**, 605.

Clement, J. J., Gorman, M. S., Wodinsky, I., Catane, R. & Johnson, R. K. (1980) Enhancement of antitumor activity of alklyating agents by the radiation sensitizer misonidazole. *Cancer Res.*, **40**, 4165.

Gomer, C. J. & Johnson, R. J. (1979) Relationship between misonidazole toxicity and core temperature in C3H mice. *Radiat. Res.*, **78**, 329.

Hall, E. J. & Roizin-Towle, L. (1975) Hypoxic sensitizers: Radiobiological studies at the cellular level. *Radiology*, **117**, 453.

Kallman, R. F., Silini, G. & van Putten, L. M. (1967) Factors influencing the quantitative estimation of the *in vivo* survival of cells from solid tumors. *J. Natl Cancer Inst.*, **39**, 539.

Rose, C. M., Millar, J. L., Peacock, J. H. & Stephens, T. C. (1980) The effect of misonidazole on *in vivo* tumour cell kill in Lewis lung carcinoma treated with melphalan or cyclophosphamide. In *Radiation Sensitizers—Their Use in the Clinical Management of Cancer*. Ed. Brady. New York: Masson. p. 250.

Stratford, I. J., Adams, G. E., Horsman, M. R. & 4 others (1980) The interaction of misonidazole with radiation, chemotherapeutic agents, or heat. *Cancer Clin. Trials*, **3**, 231.

Tannock, I. F. (1980) The *in vivo* interaction of anti-cancer drugs with misonidazole and metronidazole: Cyclophosphamide and BCNU. *Br. J. Cancer*, **42**, 871.

Twentyman, P. R., Kallman, R. F. & Brown, J. M. (1979) The effect of time between X-irradiation and chemotherapy on the growth of three solid mouse tumours: I. Adriamycin. *Int. J. Radiat. Oncol. Biol. Phys.*, **5**, 1255.

Twentyman, P. R., Brown, J. M., Gray, J. W., Franko, A. J., Scoles, M. A. & Kallman, R. F. (1980) A new mouse tumor model system (RIF-1) for comparison of end-point studies. *J. Natl Cancer Inst.*, **64**, 595.

van Putten, L. M. & Kallman, R. F. (1968) Oxygenation status of a transplantable tumor during fractionated radiation therapy. *J. Natl. Cancer Inst.*, **40**, 441.

Wasserman, T. H., Phillips, T. L., Ross, G. & Kane, J. L. (1981) Differential protection against cytotoxic chemotherapeutic effects on bone marrow CFU/s by WR 2721. *Cancer Clin. Trials.* **4**, 3.

Yuhas, J. M. (1979) Differential protection of normal and malignant tissues against the cytotoxic effects of mechlorethamine. *Cancer Treat. Rep.*, **63**, 971.

Yuhas, J. M. (1980*a*) On the potential application of radio-protective drugs in radiotherapy. In *Radiation-Drug Interactions in Cancer Management*. Ed. Sokol. New York: Wiley & Sons.

Yuhas, J. M. (1980*b*) Active *versus* passive absorption kinetics as the basis for selective protection of normal tissues by S-2-(3-aminopropylamino)-ethyl phosphorothioic acid. *Cancer Res.*, **40**, 1519.

Yuhas, J. M. & Culo, F. (1980) Selective inhibition of the nephrotoxicity of cis-dichlorodiammine-platinum by WR 2721 without altering its anti-tumor properties. *Cancer Treat. Rep.*, **64**, 57.